# Voluntary Hospitals and Municipal Authorities

**Published:** 1924-07

**Authors:** F. N. Kay Menzies


### VOLUNTARY HOSPITALS AND MUNICIPAL AUTHORITIES.

### THEIR FUTURE RELATIONS.

By F. N. KAY MENZIES, M.D., F.R.C.P., &c.

Dr. Kay Menzies began his paper with a sketch of the extent to which provision is at present made for the treatment of disease in London, in which he expressed his belief that at least four-fifths of the treatment available for the sick in the metropolitan area is provided for out of rates and taxes. He added :—I do not think there can be any doubt that these services are certain to expand, and are likely to do so in the near future in certain directions which hitherto have either not come within the scope, or only slightly so, of local authorities, for example, municipal maternity hospitals, certain diseases of childhood, such as chorea, rheumatism, infantile paralysis, etc., advanced cases of tuberculosis, etc.

### The Poor Law Infirmaries.

Future developments in the Poor Law medical service (Dr. Menzies went on) may prove to be a big factor in determining the ultimate relationships of the Voluntary Hospitals and the municipal authorities. We all know the recommendations of the Maclean Committee with regard to Poor Law Infirmaries which represent the great bulk of the institutional provision for the sick in this country. In the past they have gained a bad reputation in the minds and memories of the poorest section of the community, but it should not be overlooked (and I desire to lay great emphasis on this point) that whatever their past history may have been there is absolutely no doubt that in recent years they have taken giant strides towards making themselves thoroughly efficient hospitals, especially in London and some of the large provincial towns. Practically speaking, every Poor Law Infirmary in London to-day has upon its staff a certain number of specialists in various branches of medicine and surgery, and, moreover, they are all paid for their services. Similarly, their technical equipment is as a rule very good. Their nursing staff is better selected, better paid, better fed, better housed and works shorter hours than they used to, and it is noteworthy that some of these Poor Law Infirmaries are actually setting aside private wards and taking in private patients at fees amounting to as much as three and four guineas per week. There has, therefore, evidently been in some areas a determined effort to make them really efficient hospital units, and with the public purse behind them and a good deal of real enthusiasm for the welfare of their patients, on the part of both the Board of Management and the medical and nursing staff, there is no doubt at all that they are rapidly approaching the high standard of efficiency which hitherto has been regarded as the proud preserve of the Voluntary Hospitals.

### Rivals or Co-operators ?

If this forward movement continues, and by legislative action the " pauper taint " is removed, what do you suppose is going to be the future of these Poor Law Infirmaries ? Are they to become merely competitors with the Voluntary Hospitals or are they to work in co-operation with them ? Before answering these questions let me ask you to bear one or two points in mind. First, remember that probably the future development of the Poor Law Infirmary is in the direction of a municipal hospital ; and, secondly, that inasmuch as the local authority is already charged with the responsibility for the treatment of infectious diseases, tuberculosis, venereal disease, maternity and child welfare, school medical service, etc., as well as the organisation for the prevention of disease by means of improved housing, pure food, pure water supply, proper disposal of sewage, and all the other matters which concern the health of the community, it is almost as certain as anything can be in this world that if it can be shown that the provision of institutional accommodation for the treatment of the sick is inadequate or unsatisfactory in any area, the local authority will ultimately be compelled, whether they like it or not, by the mere force of public opinion to put it right, wherever it is proved to be required. In the present financial circumstances of the Voluntary Hospitals it is difficult to see, if any large capital expenditure be required (*vide* Cave Committee Report, page 6, paragraph 9), how they can undertake such a responsibility unless, of course, some satisfactory arrangement can be made by their boards of management with either the State or the municipality or both.

### What are the Facts ?

We must now ask ourselves what is the present position ? How far are the real needs of the public met by the various organisations, public and voluntary, which in varying degrees throughout the country purport to provide for their needs ? How far, in fact, is the problem one of an actual shortage of something which is really needed for the welfare of the community, and how far is it a problem of re-organising and grading our existing available accommodation and personnel ? Is it in fact a case of bad organisation with lack of co-ordination and over-lapping and wastage in a hundred different ways, or is it a real shortage of accommodation, equipment and finance ? I don't know, and I am perfectly certain no one else knows with any approach to accuracy. There has been in recent years a vast amount of talking and writing upon this question, and the more I hear and read of this subject the more convinced I have become that a great deal of it is ill-informed, badly advised, and not infrequently tainted by political prejudice pure and simple.

### Surveying the Situation.

The unavoidable post-war financial difficulties of the Voluntary Hospitals—the insistent and quite reasonable demand for the removal of the pauper taint from those individuals who frequently, through no fault of their own, are compelled to resort to the Poor Law Infirmaries for treatment, the immense growth in the health activities of the local authorities, as well as the introduction of the National Health

Insurance Acts—have all served to bring about a very complicated position. In short, it comes to this, that every right-thinking man and woman in this country is beginning to realise that the present position with regard to the prevention and treatment of disease, whether by voluntary or public authorities, requires review. The time has, in fact, come to make a careful, comprehensive and detailed survey and study of the situation. Such a survey would show the exact provision available from every source, voluntary, poor law, or public health. Its comprehensive character would make it possible to visualise the needs of any area, whether rural or urban, or a mixture of both. It would provide an unassailable basis upon which to build an adequate and co-ordinated scheme for the prevention and treatment of disease throughout the whole country.

### Rival Policies.

I cannot see any reason why such a scheme, when drafted, should not provide (1) for the preservation of the best features of the present voluntary hospital system ; (2) for adequate accommodation, equipment and finance of the hospitals generally ; (3) for the geographical distribution of hospitals so as to avoid unevenness and overlapping ; (4) for a closer relationship between voluntary hospitals themselves ; between voluntary hospitals and the various classes of hospitals provided by local authorities ; and between general practitioners and all the various institutions which treat the sick. In fact, it seems to me that it is only by means of such a comprehensive survey that it will be possible to give adequate consideration to the various problems concerning the sick which at present are agitating the minds of those who manage hospitals, whether voluntary or public, those who represent the medical profession, and last, but by no means least, the general mass of the community, who, after all is said and done, are the people most concerned. The Labour Party say they have made such an inquiry and as a result they have formulated a policy. Not only that, but they have taken the trouble to publish it for all the world to read. Similarly, the British Medical Association have given close attention to the same subject, and they have published the results of their deliberations and have also formulated a policy. There are other reports and other policies, some of considerable importance, to which I need not refer. If you read the views of the Labour Party and the British Medical Association and others, you will see that in certain vital matters they differ considerably, for example, in such important questions as the degree of inadequacy of institutional accommodation, on the methods by which voluntary hospitals should be subsidised, etc. Now it seems to me that in discussing a question of this kind it is absolutely essential that the data from which conclusions are drawn should be absolutely correct and therefore beyond all possible criticism.

### The Part of the Hospitals Association.

What is the British Hospitals Association going to do in view of these conflicting data and policies ? Which of them are you going to accept ? Or are you, as representing the voluntary hospitals, prepared to follow the example of those two powerful bodies, the British Medical Association and the Labour Party, and set in motion machinery to enable you to play the part to which you are entitled in this vital question ? You have done great work in the past, but circumstances have arisen which call for even greater efforts now and in the near future. It lies with you to answer certain criticisms which have been made, such as, for example, that the voluntary hospitals lack co-ordination, etc. Moreover, you must bear in mind that one of these days the Minister of Health may, in view of these very same conflicting data and policies, decide to appoint a Royal Commission to investigate the whole question. If he does, can you say that you are prepared to meet your critics armed with unassailable data and a clear and definite policy for submission to such a tribunal ? I would also beg you to keep well in your minds the fact that there is a very real danger that at some time in the near future the whole question of the treatment of the sick will be thrown into the political cockpit, and that we may have a recrudescence of that lamentable exhibition of mutual recrimination which characterised the era of the introduction of National Health Insurance in 1911. . . . The time for attacking and defending a policy from an individual or even a corporate point of view has gone. We are all heartily tired of it, and what we all most earnestly desire is a satisfactory solution of our difficulties.

### The Coming Complete Health Service.

Before concluding, I should like to emphasise my firm conviction that in this, as in many other questions of national importance, the view so frequently expressed that any survey, undertaken on the lines which I have indicated, will reveal the necessity for a vast capital expenditure, will probably prove to be erroneous. Moreover, I believe that such a survey will considerably enhance the great reputation already possessed by our voluntary hospitals and enable them to play an even greater part in the future in the work which for centuries past they have carried out for the welfare of the community. If we reflect upon the history of the prevention and treatment of disease in this country, we shall find that in its early stages the voluntary hospitals and the municipal authorities occupied limited and separate areas, leaving untouched much work of great importance to the community from the point of view of the national health. As medicine and surgery advanced and the value of institutional treatment came to be more and more fully recognised, we find the line of demarcation growing less and less distinct and the two bodies drawing gradually closer and closer together and in some cases actively co-operating to the great advantage of the health of the nation. It is impossible to suppose that the movement thus begun will stop short of anything less than a full and complete health service, available for all who require it. Therefore, I cannot too strongly impress upon you my view that the steps which you now take and the policy which you adopt in the near future will largely determine the future relationship between yourselves and the municipal authorities. Moreover, in my judgment the efficiency of the health service will in large part depend upon the establishment of intimate co-operation and harmonious relationship between the voluntary hospitals and the municipal authorities.

